# Importance of ICD-10 coding directive change for acute gastroenteritis (unspecified) for rotavirus vaccine impact studies: illustration from a population-based cohort study from Ontario, Canada

**DOI:** 10.1186/s13104-015-1412-5

**Published:** 2015-09-15

**Authors:** Sarah E. Wilson, Shelley L. Deeks, Laura C. Rosella

**Affiliations:** Public Health Ontario, 480 University Avenue, Suite 300, Toronto, ON M5G 1V2 Canada; Dalla Lana School of Public Health, University of Toronto, Toronto, ON Canada; Institute for Clinical Evaluative Sciences, Toronto, ON Canada

**Keywords:** Acute gastroenteritis, Administrative data, Epidemiology, International Classification of Diseases (ICD), Rotavirus vaccine, Vaccine impact

## Abstract

**Background:**

In Ontario, Canada, we conducted an evaluation of rotavirus (RV) vaccine on hospitalizations and Emergency Department (ED) visitations for acute gastroenteritis (AGE). In our original analysis, any one of the International Classification of Disease, Version 10 (ICD-10) codes was used for outcome ascertainment: RV-specific- (A08.0), viral- (A08.3, A08. 4, A08.5), and unspecified infectious- gastroenteritis (A09). Annual age-specific rates per 10,000 population were calculated.

**Findings:**

The average monthly rate of AGE hospitalization for children under age two increased from 0.82 per 10,000 from January 2003 to March 2009, to 2.35 over the period of April 2009 to March 31, 2013. Similar trends were found for ED consultations and in other age groups. A rise in events corresponding to the A09 code was found when the outcome definition was disaggregated by ICD-10 code. Documentation obtained from the World Health Organization confirmed that a change in directive for the classification of unspecified gastroenteritis occurred with the release of ICD-10 in April 2009. AGE events previously classified under the code K52.9, are now classified under code A09.9.

**Conclusions:**

Based on change in the classification of unspecified gastroenteritis we modified our outcome definition to also include unspecified non-infectious-gastroenteritis (K52.9). We recommend other investigators consider using both A09.9 and K52.9 ICD-10 codes for outcome ascertainment in future rotavirus vaccine impact studies to ensure that all unspecified cases of AGE are captured, especially if the study period spans 2009.

## Background

Rotavirus vaccination is a highly efficacious strategy to prevent rotavirus (RV) associated acute gastroenteritis (AGE), the most common cause of childhood AGE globally and an important contributor to childhood morbidity and mortality [1]. Two RV vaccines are licensed for use in Canada, RotaTeq^®^ (RV5, Merck) since August 2006 and Rotarix^(^™^)^ (RV1, GlaxoSmithKline) as of October 2007. Since 2008, RV vaccination has been recommended by Canada’s National Advisory Committee on Immunization [2, 3]. In August 2011, Ontario (population 13.5 million) was one of the the first Canadian jurisdiction to implement a publicly-funded program, using RV1. RV vaccines have since been included within the publicly-funded routine immunization schedules of nine of Canada’s 13 provinces and territories [4].

Numerous studies, conducted among counties of varying economic development, have demonstrated a rapid and dramatic impact of RV immunization programs, observing a reduction in the number of infants and children requiring hospitalization or Emergency Department (ED) visits by up to 85 % [5–10]. With few exceptions [11], RV infection is not typically a statutory notifiable disease and therefore not captured by routine surveillance data. Furthermore, even in countries of high economic development, most children with RV infections receive syndromic management for AGE with rehydration without laboratory confirmation [12]. Consequently, RV vaccine program impact studies have used a variety of study designs to mitigate these challenges, notably the use of administrative health services data. Such studies use a variety of outcome definitions using the World Health Organization (WHO)’s International Classification of Disease (ICD) diagnostic codes, to examine the impact of vaccination on RV-specific AGE and all cause AGE (Table 1). Although the positive predictive value is consistently high, the sensitivity of the ICD-9 [13, 14] and ICD-10 [15] codes for RV AGE (A08.0) is low and studies have included additional AGE codes to evaluate program impact, including codes which capture other viral, bacterial and parasitic pathogens and unspecified etiologies. This strategy helps to mitigate the challenges posed by misclassification of true RV infections within other diagnostic codes for AGE [13–15]. This misclassification is explained in large part by the syndromic management of AGE and variability in practice patterns for confirmation of RV as a specific causative agent [12].

**Table 1:** Examples of rotavirus vaccine impact studies conducted using ICD-10 administrative data

Study (pub year)	Country	Time period	ICD-10 codes used for outcome definition (code descriptions from WHO [20])	RV-specific AGE (A08.0) included as a discrete outcome
Jayasinghe and Macartney (2013) [15]	Australia	2000–2009	A01-A07 [excluding A02.2, A06.4, A06.5, A06.6, A06.7] (AGE of bacterial etiology) A06-A07 (AGE of parasitic etiology) A08.0 (Rotavirus AGE) A08.1-A08.4 (Other viral AGE) A09 (AGE of undetermined etiology, presumed infectious) K52 (AGE of undetermined etiology, presumed non-infectious) R198 (Other signs and symptoms involving the digestive system and abdomen)	Yes
Leino et al. (2012) [22]	Finland	1999–2005, 2010	A00-A07 (AGE due to bacterial and parasitic etiologies) A08.0 (Rotavirus AGE) A08.4 (Other viral AGE) A09 (AGE of undetermined etiology, presumed infectious) R11 (nausea and vomiting) A00.9, A01.4, A02.9, A03.9, A4.9, A05.9, A06.9, A07.9 and A09 (AGE of undetermined etiology) K52 (AGE of undetermined etiology, presumed non-infectious)	Yes
Gurgel et al. (2011) [20]	Brazil	2002–2005, 2006–2009	A08 (Rotavirus and other viral AGE) A09 (AGE of undetermined etiology, presumed infectious)	No
Molto et al. (2011) [23]	Panama	2003–2008	A00.0-A05.9 (AGE of bacterial etiology) A06.0-A07.9 (AGE of parasitic etiology) A08.0-A08.5 (Rotavirus and other viral AGE) A09 (AGE of undetermined etiology, presumed infectious)	No
Quintanar-Solares et al. (2011) [8]	Mexico	Jan. 2003–June 2009	A00-A003 (Codes capturing cholera) A04-A05 (AGE of bacterial etiology) A06.0-A06.3, A06.9 (AGE of parasitic etiologies, e.g. amoebic) A07.0-A07.2, A07.9 (AGE of parasitic etiologies, e.g. protozoal) A08 (Rotavirus and other viral AGE) A09 (AGE of undetermined etiology, presumed infectious)	No

We undertook a study of early RV vaccine program impact on hospitalizations and ED visits for AGE in Ontario, Canada using a combination of ICD-10 codes similar to the general approach used by Lopman and colleagues who used ICD-9 codes in their analyses [16]. In Canada, a country-specific modification of ICD-10 is used, ICD-10-CA [17]. Our plan was to examine program impact using both the code specific to RV AGE (A08.0) and a combination of codes capturing AGE, including RV, other viral etiologies, and AGE of undetermined infectious etiology, consistent with earlier studies. Early in our analysis, we observed an unexpected increase in AGE hospitalizations in several pediatric age cohorts beginning in April 2009. The objective of this short report is to describe our investigation of this finding, which was subsequently attributed to an ICD-10 coding directive change for AGE of undetermined etiology.

## Findings

### Methods

Data were extracted as part of a larger retrospective, longitudinal population-based cohort study to investigate the impact of a publicly-funded routine rotavirus immunization program on AGE among all Ontarians with valid provincial health insurance between the period of January 1, 2003 and March 31, 2013. There is no parallel private system for accessing health services in Ontario thus, all permanent residents in Ontario are covered under the provincial health insurance. Demographic data contained within the Registered Persons Database (RPDB) facilitated deterministic linkage across administrative databases at the individual-level using a unique identification number. These datasets were linked using unique encoded identifiers and analyzed at the Institute for Clinical Evaluative Sciences (ICES). This study was approved by the institutional review board at Sunnybrook Health Sciences Centre and Public Health Ontario in Toronto, Canada.

Individual AGE hospitalizations were obtained from the Discharge Abstract Database (DAD) of the Canadian Institutes for Health Information (CIHI) and individual ED visits from the National Ambulatory Care Reporting System (NACRS). In our original analysis, having any one of the following ICD-10-CA codes listed as the diagnosis type M, the one diagnosis or condition that can be described as being the most responsible for the patient’s hospitalization or ED visit, was used for outcome ascertainment of AGE and included: rotaviral enteritis (A08.0), other viral gastroenteritis (A08.3), viral intestinal infection, unspecified (A08. 4), other specified intestinal infections (A08.5), and other gastroenteritis and colitis of infectious and unspecified origin (A09) (definition one) (Table 2). The outcome definition was subsequently expanded to include noninfective gastroenteritis and colitis, unspecified (K52.9) (definition two). As our goal was to assess program impact on health service utilization rather than to characterize burden of disease, health services events, rather than individuals were the unit of our analysis and formed the numerator; hospitalizations and ED consultations were examined separately. For this analysis the annual age-specific population in the RPDB was used to calculate rates per 10,000 population. Data were extracted and analysed using SAS (Version 9.3).

**Table 2:** ICD-10-CA code definitions and language describing the change in coding directive

ICD-10-CA Code	Definition and notes in ICD-10-CA [26]
A08.0	Rotaviral enteritis
A08.3	Other viral gastroenteritis
A08.4	Viral intestinal infection, unspecified Includes: viral enteritis not otherwise specified (NOS), viral gastroenteritis NOS, and viral gastroenteropathy NOS)
A08.5	Other specified intestinal infections
A09	Other gastroenteritis and colitis of infectious and unspecified origin Excludes: due to bacterial, protozoal, viral and other specified infectious agents (A00-A08) and noninfective (see noninfectious) diarrhoea (K52.9)
K52.9	Noninfective gastroenteritis and colitis, unspecified Includes: diarrhea, enteritis, ileitis, jejunitis, and sigmoiditis if specified as noninfectious Excludes: colitis, diarrhoea, enteritis, gastroenteritis if coded as infectious (A09.0) or of unspecified origin (A09.9); functional diarrhoea (K59.1); neonatal diarrhoea (noninfective) (P78.3); psychogenic diarrhoea (F45.3)

Description of coding directive as described within the 2009 Canadian Coding Standards [27]	
Change	Rationale
“Deleted the directive: “Assume gastroenteritis to be noninfectious unless documented as infectious by the responsible physician”” “Added an introductory sentence: “most cases of gastroenteritis are infectious, even in industrialized countries, thus ICD-10-CA version 2009 classifies gastroenteritis NOS as infectious (A09.9 *Gastroenteritis and colitis of unspecified origin*)””	“To align with the change for v2009 ICD-10-CA. The code K52.9 *Noninfective gastroenteritis and colitis, unspecified* is assigned when specified as noninfectious”

## Results

**Fig. 1:**
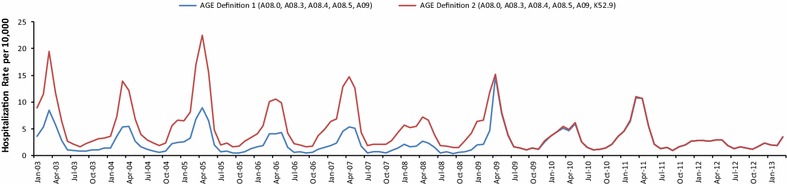
Seasonal variation in hospitalizations for acute gastroenteritis illustrated with two case definitions using ICD-10 diagnostic codes among Ontario children 0 to <24 months of age, January 2003 to March 2013

Figure 1 illustrates the characteristic seasonal variation of AGE hospitalizations and the rise in AGE among children 0 to <24 months of age occurring as of April 2009 when assessed using definition one (i.e., excluding K52.9). The average monthly rate of AGE hospitalization using this definition was 0.82 per 10,000 from period January 2003 to March 2009, rising to 2.35 over the period of April 2009 to March 31, 2013. Similar trends were found for AGE ED consultations and when the age groups assessed included children 0 to <60 months of age for both hospitalizations and ED visits (data not shown). The original outcome definition was disaggregated to examine trends by individual ICD-10-CA code. A notable rise in events corresponding to the A09 code was noted, with no increase observed for any other code within our original outcome definition. We discussed these findings with several clinical experts who were unable to provide a clinical, laboratory or administrative explanation for these observations. Australian investigators found an increase in stool testing for RV following vaccine program implementation [15] and US data suggested a modest short-term increase in number of tests performed in some laboratories [18, 19]; however, Ontario’s RV vaccine program was implemented in 2011 and the rise clearly began in 2009. Furthermore, the increase was specific to the A09 code, as opposed to across AGE codes, nor specific to the RV AGE code. Next, we corresponded with CIHI who shared documentation confirming that the rise in events corresponding to the ICD-10-CA code of A09 was explained by a change in directive for the classification of unspecified gastroenteritis contained within the release of ICD-10-CA Version 2009 which noted that “most cases of gastroenteritis are infectious, even in industrialized countries, thus the ICD-10-CA version 2009 classifies gastroenteritis not otherwise specified (NOS) as infectious (A09.9)” [20] (Table 2). The directive used earlier was deleted which read as follows: “Assume gastroenteritis to be noninfectious unless documented as infectious by the responsible physician” [20]. The date of these changes was April 1, 2009; the document containing these changes was revised further in September 2009. Therefore, cases of AGE previously classified under the code K52.9, are now classified under code A09.9 in the version of ICD-10 used in Canada (ICD-10-CA). CIHI also confirmed that the direction for this change came directly from WHO and therefore, applies to ICD-10 and all modifications of ICD-10 (i.e. ICD-10-CA).

**Fig. 2:**
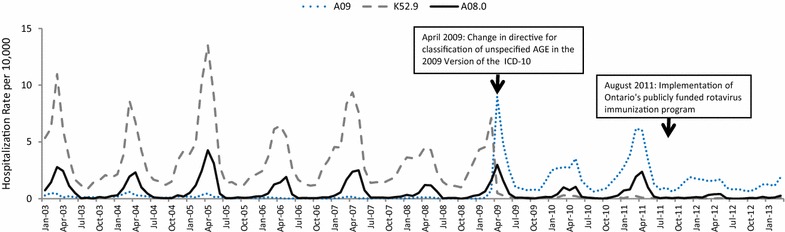
Temporal trends in hospitalizations for acute gastroenteritis, using select ICD-10-CA codes among Ontario children 0 to <24 months of age, January 2003 to March 2013

We explored the coding change within our Ontario data, adding K52.9 to our outcome definition. The impact of this modification to our outcome definition is presented in Figs. 1 and 2. Over the period of January 1 2003 and March 31 2009, the average monthly rate of hospitalizations assigned the codes captured under A09 (other gastroenteritis and colitis of infectious and unspecified origin) among children under 24 months of age was 0.12 per 10,000 population; in contrast to 1.95 per 10,000 population over the period of April 1 2009 to March 31 2013. The average monthly rate for the code K52.9 per 10,000 population was 3.62 before the coding change and 0.09 following its implementation. Further examination of the vaccine’s impact on AGE hospitalizations and ED visits in Ontario has since been conducted using the modified outcome definition.

## Discussion

Many countries have developed clinical modifications to ICD-10 in order to address their specific needs and a Canadian modification of ICD-10, ICD-10-CA, was implemented in 1995 [17]. Through our correspondence with CIHI we confirmed that the change in directive to code cases of gastroenteritis NOS as infectious, as opposed to non-infectious, was made to the ICD-10, including the ICD-10-CA in 2009. Online ICD-10 documentation from WHO for versions prior to 2010 include the note that “in countries where any term listed in A09 without further specification can be assumed to be of non-infectious origin, the condition should be classified to K52.9”; however, this statement is no longer found in the online documentation for the most current version, ICD-10 Version 2010 [21]. A small number of countries, notably including the United States (US) [22], continue to use ICD-9; although the US has used ICD-10 to code its mortality data since 1999 [22]. The coding changes described here regarding K52.9 in ICD-10 challenged the examination of trends in diarrheal deaths, in at least one US evaluation [23]. No similar change in directive for AGE classification has occurred in ICD-9. Rotavirus vaccine impact studies have utilized a wide array of ICD-10 discharge codes to capture AGE. Some have used both K52.9 and A09, while others have used only A09, to capture unspecified AGE (Table 1) [8, 9, 15, 24, 25]. The code selection for program impact studies is informed by various factors including study objectives. Future investigators need to be aware of this change in direction for AGE classification within ICD-10 when establishing their outcome definition. If the study period includes 2009, they will need to include both A09 and K52.9 to capture AGE of undetermined etiology. Given the timing of RV vaccine program introduction, inclusion of this time period is likely in many studies. In our impact study, had we excluded K52.9 from our outcome definition for AGE, we would have under-estimated program impact and instead, paradoxically found evidence of an increased burden of AGE following program implementation.

Valid estimates of RV vaccine program impact using administrative data are dependent on the comparability of hospital discharge coding practices and RV stool testing patterns pre- and post- program implementation. Most validation work of ICD codes for RV AGE has been conducted using ICD-9 in the United States, prior to program implementation [13, 14]. More recently, Jayasinghe and Macartney [15] examined hospitalization ICD-10 data and laboratory testing in a large tertiary pediatric hospital in Australia pre-and post- vaccine program implementation. They found that the sensitivity and positive predictive value of the RV-specific code (A08.0) had not significantly changed post program implementation despite evidence of greater RV stool testing following the program’s introduction [15]. Studies utilizing administrative data are important for estimating immunization program impact at a population-level; however, there are important limitations and other considerations investigators and knowledge users must be aware of. The coding directive implemented in the 2009 Version of ICD-10 is an illustrative example.

## Conclusions

The change in directive for the classification of unspecified gastroenteritis which occurred in the 2009 Version of ICD-10 is important for investigators planning evaluations of rotavirus immunization programs to be aware of. We recommend that researchers consider using both A09.9 and K52.9 to ensure that all unspecified cases of AGE, both presumed infectious and non-infectious, are captured in studies using administrative data, if the study period spans 2009.

## Abbreviations

AGE: acute gastroenteritis
CIHI: Canadian Institutes for Health Information (CIHI)
ED: Emergency Department
ICD-10: International Classification of Diseases, Version 10 (ICD-10)
NACRS: National Ambulatory Care Reporting System
RV: rotavirus
